# Evaluation of Vitamin D (25OHD), Bone Alkaline Phosphatase (BALP), Serum Calcium, Serum Phosphorus, Ionized Calcium in Patients with Mandibular Third Molar Impaction. An Observational Study

**DOI:** 10.3390/nu13061938

**Published:** 2021-06-04

**Authors:** Vito Crincoli, Angela Pia Cazzolla, Mariasevera Di Comite, Lorenzo Lo Muzio, Domenico Ciavarella, Mario Dioguardi, Maria Eleonora Bizzoca, Giuseppe Palmieri, Antonietta Fontana, Arcangela Giustino, Michele Di Cosola, Brescia Vincenzo, Roberto Lovero, Francesca Di Serio

**Affiliations:** 1 Department of Basic Medical Sciences, Neurosciences and Sensory Organs, “Aldo Moro” University of Bari, 70121 Bari, Italy; crincoli.vito@gmail.com (V.C.); mariasevera.dicomite@uniba.it (M.D.C.); 2 Department of Clinical and Experimental Medicine, Università degli Studi di Foggia, 71122 Foggia, Italy; lorenzo.lomuzio@unifg.it (L.L.M.); domenico.ciavarella@unifg.it (D.C.); mario.dioguardi@unifg.it (M.D.); marielebizzoca@gmail.com (M.E.B.); 3 Independent Researcher, 70100 Bari, Italy; joseph0794@live.it; 4 Clinical Pathology Unit, Polyclinic of Bari, 70124 Bari, Italy; a-fontana@libero.it (A.F.); bresciavincenzo58@gmail.com (B.V.); robertolovero69@gmail.com (R.L.); francesca.diserio@policlinico.ba.it (F.D.S.); 5 Department of Biomedical Sciences and Human Oncology, “Aldo Moro” University of Bari, 70121 Bari, Italy; arcangela.giustino@uniba.it

**Keywords:** vitamin D, vitamin D deficiency, alkaline phosphatase, ALPL protein, human, molar, third, mandible/growth and development, tooth eruption, tooth, impacted

## Abstract

The aim of this study was to evaluate the levels of vitamin D (25OHD) and other bone biomarkers in patients with third molar impaction (TMI). Thirty males and 30 females with unilateral or bilateral impacted mandibular third molar, and 15 males and 15 females as a control group (CG) were recruited. Rx-OPT was used to evaluate dental position and Pederson index to measure the difficulty of the intervention. Bone biomarkers were measured through blood venous sample in TMI group and CG. Mann-Whitney test, Pearson’s correlation coefficient, linear regression model were used to compare the different parameters in the two groups. 25OHD showed lower values in TMI group than in CG (*p* < 0.05) with values significantly lower in bilateral impaction (*p* < 0.05). Pearson’s coefficient for 25OHD presented a negative correlation with the Pederson index (ρ = −0.75). Bone alkaline phosphatase (BALP) showed significantly lower dosage in TMI group than CG (*p* = 0.02), Pearson’s coefficient for BALP presented a negative correlation with the Pederson index. Serum calcium, serum phosphorus, ionized calcium levels in TMI and CG groups were similar and Mann-Whitney test did not significantly differ between TMI and CG. TMI could be a sign of vitamin D deficiency and of low BALP levels that should be investigated.

## 1. Introduction

The bone turnover is regulated by various factors such as vitamin D (25OHD) while calcium and phosphorus are responsible for ensuring the maintenance of mineral homeostasis [1].

Vitamin D3 is a fat-soluble secosteroid mainly produced by UV irradiation and introduced in minimal percentage by exogenous sources (foods and supplements). It is known for being responsible for (i) the intestinal absorption of calcium, (ii) regulation of the balance calcium/phosphorus, (iii) bone growth and remodeling, (iv) tooth development [2].

Vitamin D3 increases osteoblasts’ survival and matrix maturation [3], regardless of calcium supplementation in the diet [4], promotes protein synthesis, like osteocalcin, essential for bone homeostasis [5].

25OHD reaches most of the organs through the circulatory system and acts on several cell types, thanks to the presence of vitamin D receptors (VDR) [2,6].

In the intestine, receptors bind to 1.25-dihydroxyvitamin D to stimulate calcium absorption. In the bone VDR are expressed in osteoblasts, osteocytes and osteoclasts. Recent researchers [7,8] showed that osteoblasts, through VDR expression, mediate vitamin D’s action on osteoclast activities through the RANKL/osteoprotegerin (OPG) regulatory system and that vitamin D has a direct action on osteoclasts to regulate osteoblast mineralization.

Vitamin D plays a leading role in craniofacial development and in the maintenance of good oral health [9].

During dentin formation, VDR are diffusely present in subodontoblastic cells [10]. VDR expression in ameloblasts and odontoblasts appears to be induced by vitamin D, suggesting that these cells are target cells for vitamin D [10].

There is strong evidence to support the association between vitamin D and dental-oral health [11,12,13,14]. Inadequate vitamin D concentrations have been linked to periodontal disease, higher during pregnancy [15], tooth and oral bone loss, atrophy of the salivary glands [16].

Episodes of malnutrition and vitamin D deficiency during periods of primary and permanent tooth formation can result in enamel hypoplasia, dentin/enamel hypo calcification and dental caries [17,18,19] because of the metabolic insult to ameloblasts.

Vitamin D supplementation has been shown both to reduce the relative risk for caries [20] and to improve periodontal diseases by influencing the production of antimicrobial peptide (cathelicidin and defensins), efficacious against gram-negative, gram-positive bacteria and *Candida albicans* [21,22,23].

While the US National Academy of Medicine has set the adequacy threshold at 50 nmol/L [24], there is a growing consensus that levels at or above the threshold of 75 nmol/L offer significant advantages and protection against many health disorders [25,26,27,28,29].

Calcium is the main mineral in bones and it performs various biological functions: (i) bone mineralization with the formation of hydroxyapatite, (ii) a reservoir for maintaining blood calcium levels in a physiological range, (iii) extra and intracellular signaling, (iv) cell-to-cell adhesion, (v) cell-to-cell communication, (vi) the transmission of nerve impulses and muscle contraction [30,31].

Current dietary guidelines suggest a calcium intake of 1000 to 1500 mg/day, depending on age [30,31,32].

Serum calcium levels are controlled by an integrated hormonal system that regulates calcium transport from the gut, kidneys, and bones. Parathyroid hormone PTH and the PTH receptor (PTHR), 1,25 (OH) 2D and VDR, calcitonin, ionized serum calcium and calcium-sensitive receptor (CaRare) involved in calcium metabolism [33].

Deficiency in serum calcium can affect teeth development with enamel hypo-calcification, sensitivity, high caries susceptibility, teeth mobility and loss [34].

The second most abundant mineral in bones is phosphorus. It is present in all mineralized tissues (skeletal tissues and teeth) in all cells and tissues. It performs various biological functions: (i) as an essential component of DNA, RNA and phospholipids, (ii) source of high-energy bonds in adenosine triphosphates (ATP), (iii) substrate for various kinases and phosphatase, (iv) a regulator of intracellular signaling [35].

Serum Pi levels are determined by dietary load and reabsorption of renal glomerular filtrate in the proximal tubule [35].

The appropriate levels of phosphorus are regulated by various hormones Vitamin D, Parathyroid hormone), phosphatonins (FGF23) phosphatonin-like factors (Dentin matrix protein 1 (DMP1) and other factors that act on the bone-kidney-gut axis [36,37]. The systemic and local homeostasis of Pi and inorganic pyrophosphate (PPi) influence the mineralization of the hard tissues of the oral cavity. An appropriate balance of Pi/PPi locally, coupled with physiological serum levels of Pi, is required for the development of teeth and supporting tissues [35].

Phosphate dysregulation affects tooth roots and supporting tissues: loss of bone, cementum and periodontal ligament attachment [35]. Besides it has been demonstrated that the daily dietary consumption of phosphorus in association with sugar was associated with a high salivary concentration of IL-1β and a reduced salivary concentration of IL-4 favoring the onset of oral diseases including gingivitis with dental decay in children aged 10 and over [38,39].

A study on time-mated FVB wild-type mice and fed high-calcium, low-phosphorus diets in utero up to weaning age showed morphological changes and bone mineral density differences in the jaws of offspring exposed to this experimental diet: retrognathic mandibles associated with a reduced bone mineral density in females, mandibles with height and length of the mandibular body increased in males with condyles similar to those observed in females. Altering calcium and phosphorus levels in utero affects adult mouse mandibular morphology [30,40].

An important marker of bone turnover is bone alkaline phosphatase (BALP), the bone-specific isoform of ALP (alkaline phosphatase) considered to be a highly specific marker of the bone-forming activity of osteoblasts [41,42] and exhibits an increase in levels during childhood and adolescence [43].

It is an ectoenzyme produced by the osteoblast during the matrix maturation phase and is down-regulated in calcifying osteoblasts.

In bone, BALP is essential for biomineralization, mediates the hydrolysis of pyrophosphate, releasing inorganic phosphate, which is a major component of hydroxyapatite in bone and is the favored marker for bone turnover in patients with impaired kidney function because its clearance does not depend on glomerular filtration.

Tripathi T. et al. demonstrated that BALP could be used as a potential biomarker to estimate skeletal and mandibular growth status with a peak at the beginning of stage 3 CVMI (cervical vertebral maturation index according to Franchi and Baccetti) and after progression a year, a descent into the advanced stage 3 [44].

The literature lacks studies about the association between vitamin D, serum calcium, ionized calcium, phosphorus, and BALP levels and mandibular third molar impaction.

The mandibular third molar is the more frequently impacted tooth, affecting about 20–30% of the population [45,46], followed by the upper third molars and the upper canines. The etiology could depend on different factors: (i) a short distance from the distal surface of the lower second molar to the branch [47], (ii) amount and direction of mandibular growth [48], (iii) width and remodeling of the branch [49], (iv) degree of the third molar maturity [50], (v) inclination of posterior teeth [51].

A different amount of branch resorption has been shown to influence the direction of condylar growth, thus affecting the morphology, position of the adult mandible and the eruptive path of the lower third molar. Poor resorption in the anterior part of the branch and a mandibular forward rotation causes condylar growth in a predominantly vertical direction. In contrast, a more generous amount of resorption with a backward rotation of the jaw makes condyles grow backward. Molars tend to erupt further during the functional phase in patients with anterior growth rotation, partially compensating for the limited amount of resorption at the branch’s anterior edge. Another explanation for mandibular third-molar impaction might be an unfavorable path of eruption. Typically, the tooth bud is mesially tilted during the initial stages of calcification and root development [52]. Longitudinal evaluations show that on average, in subjects who have not been treated orthodontically, third molar elevation usually occurs in early adolescence. However, it is not uncommon to have non-uprighting during adolescence. The combined rate of mesial and horizontal occlusions of approximately 46% suggests that failed molar lift may be a common cause of occlusion [52].

A decrease of the mechanical stresses targeting on the jaw during mastication due to artificial feeding of infants and excessive consumption of sweet and soft food by children and youth have also been reported [53,54,55].

Besides, dental impaction could be due to systemic factors, such as incorrect nutrition, anemia, specific infections such as syphilis and tuberculosis) rare diseases, syndromes (cleidocranial dysplasia, oxycephaly, progeria, achondroplasia) [56,57,58,59,60,61,62,63].

Given this background, this study aimed to evaluate the levels of vitamin D, serum calcium, ionized calcium, phosphorus, and BALP, main biomarkers required routinely to assess bone turnover, in patients with third molar impaction (TMI).

## 2. Materials and Methods

This observational study was conducted from October 2019 to April 2020 in accordance with The Code of Ethics of the World Medical Association (Declaration of Helsinki) for experiments involving humans at the Dental Clinic of the University of Foggia and the Odontostomatology Clinic of the University of Bari. It was decided to conduct the study in this period to avoid seasonal variation in 25OHD levels. Sixty patients (30 men and 30 women) were recruited in the study group. The study was approved by the ethics committee of Bari (Italy) (N. 6481 VIT.D-BALP INCLUSION protocol number 0054625/31-07-2020) and informed consent was obtained.

Inclusion criteria were: (i) individuals of both sexes, age > 18 years, (ii) Caucasian ethnic origin, (iii) patients who have not changed their lifestyle (work, sport, hobbies), food and drug intake (vitamin D and calcium supplements), sun exposure, socio-demographic and anthropometric factors in the last 6 months [64], (iv) unilateral or bilateral mandibular third molar impaction (TMI).

Exclusion criteria were: (i) traumatic diseases (trauma and fractures of the maxillofacial region and other parts of the body), (ii) missing teeth, (iii) head, oral or neck neoplasia, (iv) past or present chemotherapy and radiotherapy involving head, neck and any other area of the body, (v) neurological disorders, (vi) maxillofacial treatments (tumors and orthognathic surgeries), (vii) pregnancy. Patients affected by acute and chronic systemic disease (Crohn disease, ulcerative colitis, malabsorption syndromes, diabetes, osteoporosis, renal insufficiency, hormonal diseases, thyroid pathologies) were excluded, as well as smokers and people with alcohol or drug dependence. Patients under steroid or bisphosphonate treatment under monoclonal antibodies or vitamin D therapy were also not included. Patients’ age ranged between 18 and 59 years, with a mean age of 28.5 ± 8.88 years in the study group. A control group (CG) of 30 subjects (15 men and 15 women) with a mean age of 28.06 ± 8.02, matched by age with the study group, was randomly chosen among those presenting lower third molars in a correct position, without any problem of eruption. In the control group the mean age of eruption of the third molars was 21 ± 4 (Table 1a). In the TMI group, 76.6% had unilateral third molar inclusion, while 23.4% bilateral (Table 1b).

A single experienced practitioner assessed dental status through an anamnestic questionnaire and a clinical examination and Rx-OPT was required. The position of wisdom teeth was determined using Winter’s classification and Pell and Gregory’s classification based on OPT [65,66].

The Winter scheme is based on the inclination of the impacted third molar with respect to the long axis of the second molar (Table 2a).

According to Pell and Gregory, third molars are classified in Class I, II, or III, according to their relationship with the ascending mandibular ramus (the space available distally to the second molar). Moreover, they also consider three different levels—A, B, and C—according to the depth of the impacted tooth in the bone with respect to the occlusal plane (Table 2b).

According to the Pederson index, the sum of these two classifications gives a score of intervention difficulty (Table 2c).

A venous blood sample was taken at the same time of the first dental examination to measure 25OHD values, BALP, serum calcium, serum phosphorus, and ionized calcium to evaluate each patient’s mineral balance. Blood from the forearm vein was collected into 5-mL Vacutainer tubes with no anticoagulant. The blood samples were centrifuged (1000× *g*, 15 min, 4 °C), serum was removed and immediately stored at −80 °C until analyzed. The dosage of vitamin D was performed with chemiluminescence using the LIAISON^®^XL (DiaSorin, Vercelli-Italy) instrumentation. The dosage of BALP was performed with chemiluminescence assay using the TGSTA Technogenetics instrumentation (Technogenetics, Milano-Italy). BALP is primarily a biochemical marker of bone turnover suitable for monitoring metabolic bone disease, particularly the management of chronic kidney disease—mineral and bone disorders. The dosage of serum calcium, phosphorus, and ionized calcium was carried out using the colorimetric method on Dimension VISTA 1500 Instrumentation (Siemens).

The variables studied in the database have been: age, sex, unilateral or bilateral impaction, degree of difficulty of the extraction according to the Pederson index score obtained by Winter and Pell and Gregory classifications. 25OHD, BALP, ionized calcium, serum phosphorus, and serum calcium levels were analyzed using the Mann-Whitney non-parametric test.

To understand the links among all the variables considered, Pearson’s linear correlation coefficient was applied.

The Welch test for unequal variances was used to evaluate the difference in vitamin D dosage between the males and females’ subgroups. A linear regression model using the Student’s *t*-test was adopted to evaluate a possible association between 25OHD, BALP, ionized calcium, serum phosphorus, serum calcium levels, and the Pederson index.

The variables with a significance greater than 5% were maintained, obtaining a model that satisfactorily explains the phenomenon studied.

The Shapiro-Wilk test was used to compare the linear regression model’s residues with a standard normal distribution. The Breusch-Pagan test was performed for verification of homoscedasticity.

For all the tests used, a *p*-value threshold of 5% was adopted and all processes and calculations were performed using the statistical software R (v. 3.6.1, 2019) and Prism GraphPad (v. 6.0).

## 3. Results

**Figure 1:**
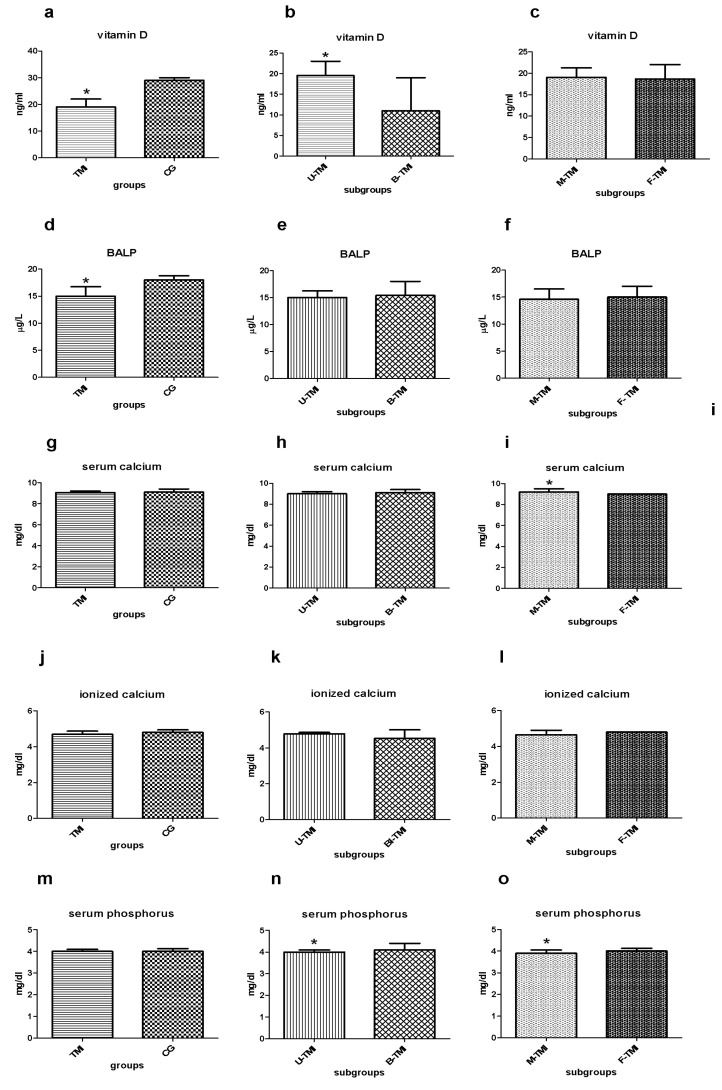
(**a**–**c**) Median with interquartile range of serum Vitamin D dosage (ng/mL) in TMI vs. CG (**a**), in U-TMI vs. B-TMI (**b**) and male vs. female TMI patients (**c**); (**d**–**f**) median with interquartile range of ionized calcium dosage (μg/L) in TMI vs. CG (**d**), in U-TMI vs. B-TMI (**e**) and M-TMI vs. F-TMI patients (**f**); (**g**–**i**) median with interquartile range of serum calcium dosage (mg/dL) in TMI vs. CG (**g**), in U-TMI vs. B-TMI (**h**) and male and female TMI patients (**i**); (**j**–**l**) median with interquartile range of ionized calcium dosage (mg/dL) in TMI vs. CG (**j**), in U-TMI vs. B-TMI (**k**) and male and female TMI patients (**l**); (**m**–**o**) median with interquartile range of ionized calcium dosage (mg/dL) in TMI vs. CG (**m**), in U-TMI vs. B-TMI (**n**) and M-TMI vs. F-TMI patients (**o**). Abbreviations CG control group, U-TMI unilateral third molar impaction, B-TMI bilateral third molar impaction, M-TMI third molar impaction for males, F-TMI third molar impaction for females; * denotes a significant statistically differences.

The dosage of 25OHD in the two groups is different (Figure 1a–c): non-parametric Mann-Whitney test showed significantly lower levels for 25OHD (ng/mL) in the TMI group than CG (median TMI = 19, CG = 29; *p* < 0.05). Within the study group, the levels of 25OHD are even lower in the case of bilateral impaction (B-TMI) (median U-TMI = 19.5, B-TMI = 11; *p* < 0.05). No statistically significant difference is found between male and female TMI patients (median M-TMI = 19, F-TMI = 18.6; *p* = 0.57). The dosage of BALP in the two groups is different (Figure 1d): the non-parametric Mann-Whitney test shows significantly lower levels (μg/L) in the TMI group than CG (median TMI = 15, CG = 18; *p* = 0.02). Within the study subgroups, the levels of BALP are similar both in the comparison between U-TMI vs. B-TMI (median U-TMI = 15, B-TMI = 15.4 *p* = 0.53) and males vs. females (median M-TMI = 14.6, F-TMI = 15 *p* = 0.98); consequently, no statistically significant differences were found (Figure 1e,f).

The dosage of serum calcium (mg/dl) in TMI and CG groups is similar. The non-parametric Mann-Whitney test does not significantly differ in the TMI group than CG (median TMI = 9, CG = 9; *p* = 0.24). Furthermore, the comparison between U-TMI and B-TMI does not reveal any difference (median U-TMI = 9, B-TMI = 9; *p* = 0.7). On the contrary, statistically significant differences are found between male and female TMI patients (median M-TMI = 9.2, F-TMI = 9; *p* < 0.05) (Figure 1g–i).

The ionized calcium levels (mg/dl) are similar in the groups and subgroups considered. Mann-Whitney test does not show significantly differences (median TMI = 5, CG = 5, *p* = 0.08; median U-TMI = 5, B-TMI = 5, *p* = 0.32; M-TMI = 5, F-TMI = 5, *p* = 0.83) (Figure 1j–l).

Serum phosphorus (mg/dl) in TMI and CG shows perfectly identical levels and the non-parametric Mann-Whitney test does not significantly differ between the two groups (median TMI = 4, CG = 4; *p* = 0.29). Conversely, the comparison between U-TMI and B-TMI reveals a slightly higher dosage of serum phosphorus in B-TMI patients (median U-TMI = 4, B-TMI = 4.1; *p* = 0.03). A similar trend has been found in comparing male and female patients (median M-TMI = 3.9, F-TMI = 4; *p* ˂ 0.05). Into the subgroups, the statistical Mann-Whitney test reveals significant differences in both cases (Figure 1m–o).

**Figure 2:**
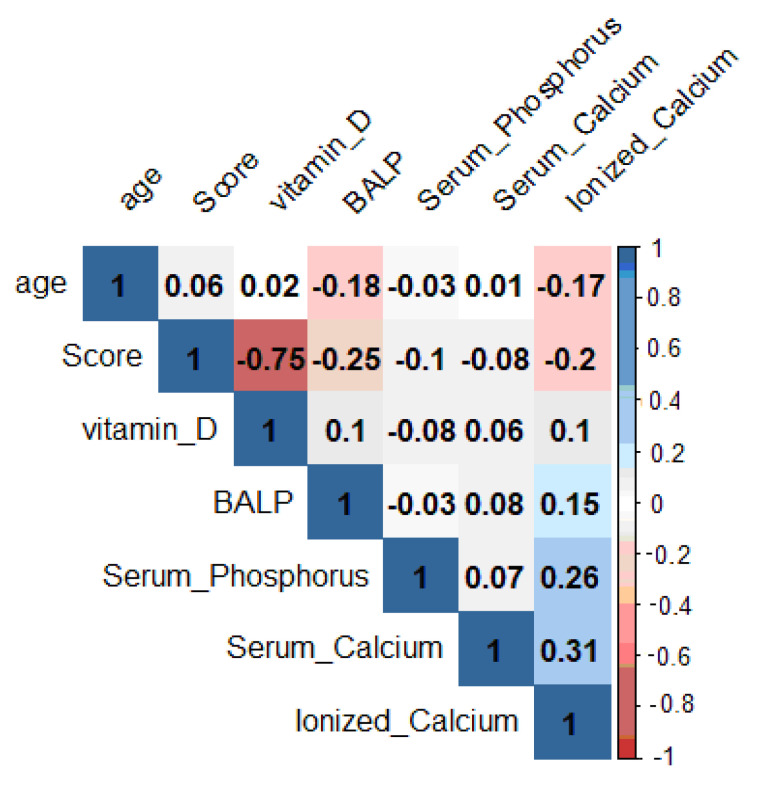
Evaluation of the correlation coefficients of the parameters taken into consideration.

Pearson’s correlation shows the statistical relationship between pairs of the continuous variables examined, thus giving information about the magnitude of the association and the direction of the relationship. In the ladder chart (Figure 2), coefficient values can range from +1 to −1, where +1 indicates a perfect positive relationship, −1 indicates a perfect negative relationship and a 0 indicates no relationship exists (Table 3) [67].

25OHD shows a linear correlation coefficient inversely proportional to the Pederson index (ρ = −0.75). BALP showed an inverse correlation (ρ = −0.25) versus score, although less markedly than 25OHD. The relationship between score and calcium serum, phosphorus serum and ionized calcium is not significant.

**Figure 3:**
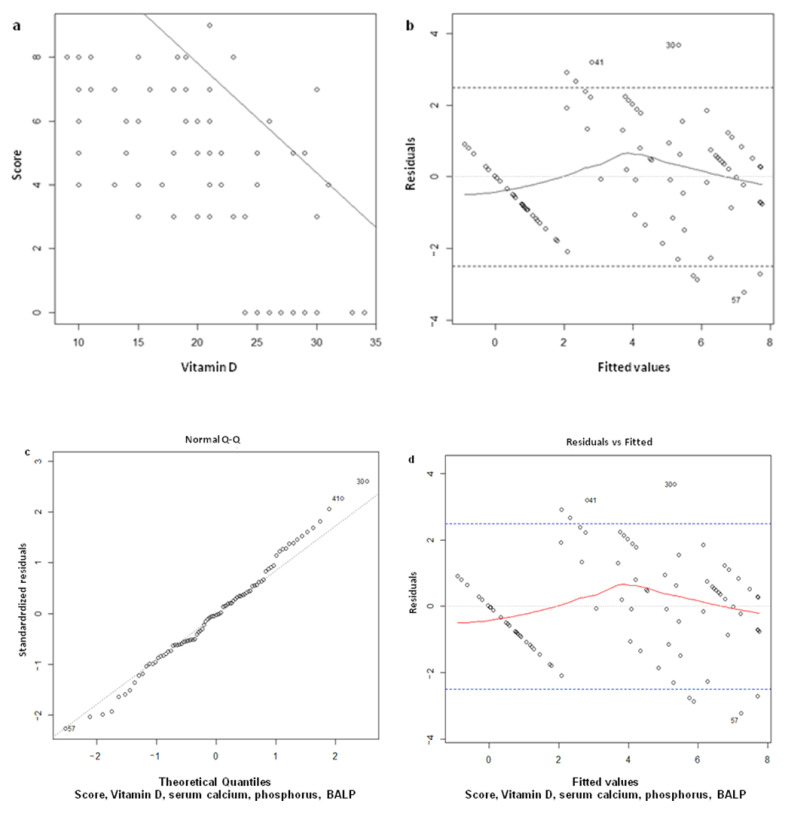
(**a**) Regression line score vs. vitamin D; (**b**) verification of hypotheses of linearity by studying the residuals with respect to the theoretical values of the model. The graph shows a slight asymmetry around 0, which could be considered acceptable in view of the number of samples; (**c**) verification of the residues’ normality, comparing those utilized in the model to a standard normal distribution. The hypothesis is fully respected and verified by the Shapiro-Wilk test (*p*-value = 0.83); (**d**) verification of homoskedasticity, comparing the square root of the standard residuals to the model’s values. Although the graph does not show an ideal situation, homoskedasticity was verified by the Breusch-Pagan test (*p*-value observed > 0.05).

The study of the relationship between score and 25OHD showed a straight line representing the linear link between the two variables, decreasing with increasing 25OHD (Figure 3a). Therefore, this data confirms a linear correlation coefficient equal to −0.75 between 25OHD and score.

The demonstration that the model’s erratic values do not deviate too much from the range of tolerance to the variation of the theoretical values obtained by the model (fitted values) has been reported in Figure 3b. The model has also been validated by checking with Shapiro-Wilk’s test the residual normality (erratic component) and shows that the hypothesis has been respected (*p* = 0.83) (Figure 3c). Finally, the demonstration that the square root of the model’s erratic values does not deviate from the range of tolerance to the variation of the theoretical values obtained from the model’s mathematical relationship (fitted values) has been reported in Figure 3d.

## 4. Discussion

The third molars are the last to erupt with a relatively high probability of being impacted.

Various hypotheses have been elaborated in the literature for the impaction of the mandibular third molar: (i) insufficient development of the retromolar space due to progressive evolutionary reduction in the size of the jaw [53]; systemic factors such as incorrect nutrition (vitamin deficiencies), dysmetabolic conditions, endocrine disorders and infectious diseases [68,69].

Relatively of “the stomatognathic system”, Mona et al. suggested that supplementation of vitamin D combined with an antiresorptive agent (calcitonin) may enhance mandibular growth and potentially produce a beneficial effect on orthodontic treatment for stunted mandibular bone growth problems [70].

It has been demonstrated in rats, through a radiographic and cephalometric technique, how a diet lacking in vitamin D and calcium can causes disturbances to the osteogenesis in the growth sites, with relevant consequences on the growth and shape of the viscerocranium and neurocranium [71].

Vitamin D plays a more dominant role in mandibular alveolar bone and dental formation than PTH because of its lesser sensitivity to endogenous PTH’s anabolic actions than long bones [72].

Besides, evidence reveals that vitamin D interferes with bone resorption processes during tooth eruption due to its direct induction of osteoblasts-mediated osteoblastogenesis. The eruption phenomenon requires the participation of osteoclasts that intervene in the process of reabsorption of the alveolar bone, mainly mediated by the expression of the colony-stimulating factor 1 (CSF-1), of the RANK, the RANK ligand (RANKL) and of the osteoprotegerin (OPG). The RANKL/RANK/OPG system is considered the path that modulates bone remodeling and can control the dental rash [73,74,75].

Vitamin D improves the osteoclast-inductive capacity by increasing the RANKL/OPG ratio [76] and transactivates the gene RANKL predominantly via the RUNX2-dependent pathway, but also via a RUNX2-independent mechanism that depends on direct binding of the VDR complex to the VDRE element in the RANKL promoter [77,78].

Vitamin D deficiency in association with the RUNX2 gene mutation, that is involved in bone remodeling, tooth formation and eruption, might be the cause of multiple dental inclusions [78] in patients with cleido-cranial-dysplasia in which skeletal anomalies, short stature, osteopenia, and osteoporosis are also present [79].

To date, no studies have investigated vitamin D levels in patients with mandibular third molar impaction. Only Oteri et al. have correlated the degree of difficulty in wisdom tooth extraction and related complications to the vitamin D dosages. By administrating 300,000 IU of cholecalciferol four days before the surgical removal of lower third molars, a significant reduction of edema post-surgery and inflammatory responses leading to a more favorable clinical course were reported [80].

A lower incidence of postoperative infections after oral or intramuscular administration of antibiotics was also demonstrated [81].

In this observational study, the significant decrease of 25OHD level in the TMI group than CG (*p* < 0.05) testifies a probable involvement of vitamin D in the impaction of the lower third molar. This hypothesis is also supported by the presence of significantly lower values in the group of patients with bilateral inclusion conditions (B-TMI) (*p* < 0.05). This observation suggests that vitamin D could influence mandibular growth with inadequate development of the retromolar space.

The value of the Pearson’s coefficient (ρ = −0.75) shows a negative correlation between the Pederson index and serum concentration of 25OHD. The data from this study reveal that males and females are equally affected in the TMI (*p* = 0.57). Moreover, the individuals’ age does not show any marked correlation with the parameters studied (ρ = −0.06).

Similar to what was found for 25OHD, the dosage of BALP (Figure 2a) also shows significantly lower levels in the TMI group than CG (*p* = 0.02) and the value of the Pearson’s coefficient (ρ = −0.25) shows a negative correlation with the Pederson index again, although it is weaker. These results show an inverse relationship between the difficulty in extracting the lower wisdom tooth and BALP levels and confirm literature data showing a correlation negative between BALP and mandibular length across CVMI stages 3 to 5. Besides, our results, in accordance with literature data, demonstrated a link between the activity of vitamin D and BALP. In particular, it has been reported that vitamin D induces ALP activity and mineralization of human dental pulp cells and human dental follicle cells in basal cell culture medium without osteogenic factors [82,83,84].

Finally, the BALP dosage does not show significant variations between U-TMI e B-TMI subgroups, and it seems not to change with the sex of the patients.

In this study, the dosage of 25OHD and BALP has been put beside by quantitative evaluation of serum calcium, ionized calcium, and serum phosphorus because disorders of mineral metabolism could be directly related to vitamin D and parathyroid hormone (PTH) alterations. In particular, a decrease in ionized calcium stimulates a release of PTH, which maintains calcium homeostasis by increasing bone mineral dissolution, thus releasing calcium and phosphorus, by increasing renal reabsorption of calcium and excretion of phosphorus, and by enhancing the gastrointestinal absorption of both calcium and phosphorus indirectly through its effects on the synthesis of Vitamin D [85].

The dosage of serum calcium and ionized calcium in TMI and CG groups is similar and the non-parametric Mann-Whitney test does not show any significant difference. Furthermore, also the comparison between U-TMI and B-TMI did not reveal any difference. Statistically significant differences were found between male and female TMI patients (*p* < 0.05) for serum calcium in accordance with the literature [86].

The dosage of serum phosphorus is similar in TMI and CG and the non-parametric Mann-Whitney test does not show any significant difference between the two groups. Conversely, the comparison between U-TMI and B-TMI revealed a slightly higher dosage of serum phosphorus in B-TMI patients (*p* = 0.03). A similar trend was found in the comparison between male and female patients (*p* ˂ 0.05) in discordance with literature due to the sample’s age inhomogeneity [87].

Our results showed lower levels of vitamin D and BALP in patients with TMI.

Mandibular third molar dysodontiasis could be considered a sign of low level of BALP and vitamin D deficiency. This suggests investigating for the deficit of vitamin D in the presence of third molar impaction considering the association between lower serum 25OHD levels, a major public health problem worldwide, and various infectious diseases including COVID-19 [88], cancer, autoimmune and dermatological diseases.

## 5. Conclusions

This pilot study is the first that investigated vitamin D, serum calcium, ionized calcium, phosphorus and BALP levels in patients with third mandibular molar impaction (TMI) and it showed that mandibular third molar impaction could be considered a predictive sign of vitamin D deficiency. Therefore, it is necessary to investigate the levels of bone bio-markers and especially of vitamin D in the presence of disodontiasis of the mandibular third molar. Further clinical and experimental validations with broader statistical analysis and larger samples will be needed to confirm the hypothesis of this pilot study.

## Figures and Tables

**Table 1:** **a.** Demographic characteristics of the Third Molar Impaction (TMI) group and controls **b.** Type of third molar impaction in the TMI group and male/female subgroups.

**a. Demographic Characteristics of the TMI Group and Controls**			
**Demographic Characteristics (Mean, ±SD)**	**TMI**	**CG**	
Age	29 ± 9	28± 8	
Men age	29 ± 11	28 ± 11	
Female age	27 ± 6	28 ± 9	
**b. Type of Third Molar Impaction in the TMI Group and Male/Female Subgroups.**			
**Type of impaction (n, %)**	**TMI**	**Male**	**Female**
Unilateral	46 (77%)	22 (73%)	24 (80%)
Bilateral	14 (23%)	8 (27%)	6 (20%)

**Table 2:** **a.** Winter classification, **b.** Pell and Gregory’s classification, and **c.** Pederson index: difficulty score is the sum of the score relating to Winter’s and Pell and Gregory’s classifications.

**a. Winter Classification**		
**Type**	**Description**	**Score**
Mesioangular	Long axis of the 3rd molar inclined in mesial direction parallel to the 2nd molar.	1
Horizontal	Long axis of the 3rd molar perpendicular to the 2nd molar.	2
Vertical	Long axis of the 3rd molar parallel to 2nd molar.	3
Distoangular	Long axis of the 3rd molar inclined in distal direction to 2nd molar.	4
Inverted	Crown of the 3rd molar directed to basilar of the mandible.	5
**b. Pell and Gregory’s Classification**		
**Type**	**Description**	**Score**
Class I	There is sufficient space between the ramus and the distal part of the 2nd molar for the accommodation of the mesiodistal diameter of the 3rd molar.	1
Class II	The space between the 2nd molar and the mandible ramus is less than the mesiodistal diameter of the 3rd molar.	2
Class III	All or most of the 3rd molar is in the ramus of the mandible.	3
Position A	The occlusal plane of the impacted tooth is the same level as the occlusal plane of the 2nd molar.	1
Position B	The occlusal plane of the impacted tooth is between the occlusal plane and the cervical line of the 2nd molar.	2
Position C	The impacted tooth is below the cervical line of the 2nd molar.	3
**c. Pederson Index: difficulty score is the sum of the score relating to** **The Winter and Pell & Gregory’s classifications.**		
**(Difficulty Score)**		**Total**
1. easy		3–4
2. moderate		5–6
3. difficult		7–10

**Table 3:** Guide to interpretation of the correlation coefficient.

Correlation Coefficient	Medicine (Chan YH)
+1 −1	Perfect
+0.9 −0.9	Very Strong
+0.8 −0.8	Very Strong
+0.7 −0.7	Moderate
+0.6 −0.6	Moderate
+0.5 −0.5	Fair
+0.4 −0.4	Fair
+0.3 −0.3	Fair
+0.2 −0.2	Poor
+0.1 −0.1	Poor
0 0	None

## Data Availability

The data presented in this study are available on request from the corresponding author upon reasonable request.
